# Dataset size and composition impact the reliability of performance benchmarks for peptide-MHC binding predictions

**DOI:** 10.1186/1471-2105-15-241

**Published:** 2014-07-14

**Authors:** Yohan Kim, John Sidney, Søren Buus, Alessandro Sette, Morten Nielsen, Bjoern Peters

**Affiliations:** La Jolla Institute for Allergy & Immunology, 9420 Athena Circle, La Jolla, CA 92037 USA; Department of International Health, Immunology and Microbiology, University of Copenhagen, Blegdamsvej 3, 2200 København N, Copenhagen, Denmark; Center for Biological Sequence Analysis, Department of Systems Biology, The Technical University of Denmark, Building 208, Lyngby, DK-2800 Denmark; Instituto de Investigaciones Biotecnológicas, Universidad Nacional de San Martín, San Martín, Buenos Aires, B 1650 HMP Argentina

**Keywords:** Benchmarking of MHC class I predictors, Epitope prediction, Sequence similarity, Cross-validation

## Abstract

**Background:**

It is important to accurately determine the performance of peptide:MHC binding predictions, as this enables users to compare and choose between different prediction methods and provides estimates of the expected error rate. Two common approaches to determine prediction performance are cross-validation, in which all available data are iteratively split into training and testing data, and the use of blind sets generated separately from the data used to construct the predictive method. In the present study, we have compared cross-validated prediction performances generated on our last benchmark dataset from 2009 with prediction performances generated on data subsequently added to the Immune Epitope Database (IEDB) which served as a blind set.

**Results:**

We found that cross-validated performances systematically overestimated performance on the blind set. This was found not to be due to the presence of similar peptides in the cross-validation dataset. Rather, we found that small size and low sequence/affinity diversity of either training or blind datasets were associated with large differences in cross-validated vs. blind prediction performances. We use these findings to derive quantitative rules of how large and diverse datasets need to be to provide generalizable performance estimates.

**Conclusion:**

It has long been known that cross-validated prediction performance estimates often overestimate performance on independently generated blind set data. We here identify and quantify the specific factors contributing to this effect for MHC-I binding predictions. An increasing number of peptides for which MHC binding affinities are measured experimentally have been selected based on binding predictions and thus are less diverse than historic datasets sampling the entire sequence and affinity space, making them more difficult benchmark data sets. This has to be taken into account when comparing performance metrics between different benchmarks, and when deriving error estimates for predictions based on benchmark performance.

**Electronic supplementary material:**

The online version of this article (doi:10.1186/1471-2105-15-241) contains supplementary material, which is available to authorized users.

## Background

Major Histocompatibility Complex (MHC) molecules belong to a large family of proteins used by the immune system to recognize foreign antigens such as pathogens. In humans, MHCs are called human leukocyte antigens (HLA). Attached to the cell-surface, MHC molecules loaded with peptide fragments of intra- or extra-cellular origin are presented to T-cells for recognition, after which cell-killing or downstream signaling events are triggered [1]. Hence, binding of peptides to MHC molecules is a requirement for T-cell recognition [2, 3]. Accordingly, accurate peptide:MHC binding predictions are useful for the development of reagents, therapeutics and diagnostics for infectious and autoimmune diseases, allergy and cancer.

Because of the importance of peptide:MHC binding in determining T-cell epitopes, much effort has been expended to collect experimentally measured binding affinity data and make them available to the scientific community [4–8]. Accompanying the growth of the binding data, many MHC class I peptide binding predictors have been reported to date. To compare their predictive performances, a number of large-scale benchmarking studies have been carried out. In the case of MHC-I predictors, high predictive performances with average Areas under Receiver Operating Characteristic curves (AROCs) of ~0.9 from cross-validations have been reported [9, 10], suggesting that the predictive methods have matured.

Despite much progress in the development of predictive methods for binding of peptides to MHC class I molecules, a number of important questions remain. First, given that cross-validated predictive performances are *estimates* of ‘true’ performances in real world applications, how accurate are these estimates? Second, what is the role of sequence similarity in influencing the accuracies of these estimates? That is, does presence of similar peptides between testing and training sets lead to inflated predictive performances? Third, are there additional factors that lead to deviations between blind and cross-validated performances?

To address these questions, we tested existing predictive methods against a large set of blind data sets and measured deviations of cross-validated performances with respect to those on a blind dataset. We introduced a cross-validation strategy where sequence similarity between testing and training data sets is dramatically reduced. Furthermore, we examined various characteristics of cross-validation and blind data sets to better understand how they influence estimates of blind predictive performances.

## Results

### Assembling a comprehensive set of MHC class I binding data for cross-validations

To better understand the factors that contribute to accuracy of predictive performances estimated by cross-validation, we prepared the three binding data sets shown in Table 1. The binding data came from the Immune Epitope Database (IEDB) [8], as well as some data from submissions currently in process from the Buus and Sette labs. *BD2009* and *BD2013* are data sets prepared in years 2009 and 2013, respectively, for re-training of the predictive tools hosted on the Immune Epitope Database Analysis Resource (IEDB-AR) [11]. Cross-validated predictive performances were generated against *BD2009*. Compared to *BD2009*, *BD2013* contained about 30% more data points. The *BD2013* data set covered 6 species (including human, mouse, and macaque), 114 MHC-I alleles, 257 (MHC, length) data sets with 685 affinity measurements on average, and a total of 176,161 measurements. *BD2013* is the largest binding affinity measurement data set assembled to date for MHC class I peptide binding.

**Table 1:** **Binding data statistics**

	BD2009 ^+^	BD2013	Blind ^++^
**Alleles**	79	114	53
**Data sets**	170	257	90
**Data set size**			
Average	792	685	324
Min	50	50	50
Max	6,961	8,826	1,865
**Total data points**	134,645	176,161	29,169

^+^All cross-validations were carried out using *BD2009*.

^++^ *Blind* was generated by subtracting *BD2009* from *BD2013*. Against *Blind*, all blind predictions were made using the predictors trained on *BD2009*.

Each (MHC, length) combination is associated with a data set.

To prepare independent data sets against which to estimate ‘true’ predictive accuracy, we compiled a blind data set (i.e. ‘*Blind’)*. Against this data set, all blind predictions used in this study were generated with the predictors trained on *BD2009*. The *Blind* set was prepared by subtracting *BD2009* from *BD2013* and removing ‘similar’ peptides with respect to *BD2009*. Two peptides were considered ‘similar’ if they shared at least 80% sequence identity and were of same lengths. In the table, only those data sets for which (MHC, length) combinations were shared between *BD2009* and *Blind* and that had at least 50 data points are reported. The *Blind* data set contained binding data associated with 53 alleles, 90 (MHC, length) data sets, and 29,160 measurements. All of the benchmark data sets mentioned are available at the IEDB-AR benchmark datasets website [12].

### Cross-validations tend to over-estimate blind predictive performances

In a typical run of cross-validation, a data set is randomly partitioned into *N* subsets, and one subset is held out for making predictions using a predictor trained on the remaining *N - 1* subsets. This type of cross-validation has been used widely for benchmarking peptide:MHC binding predictors [9, 10, 13]. To distinguish it from other types of cross-validations that will be introduced later, we will call it *cv_rnd* from here on, indicating a random partitioning for cross-validation.

In terms of absolute predictive performances against the *cv_rnd* cross-validation data sets, NetMHCpan performed better than either SMM^PMBEC^ or NetMHC, while SMM^PMBEC^ and NetMHC performed similarly (similar rank of the three methods was found for the other two data cv data sets, for details see in Additional file 1: Table S1 ). Against the blind data sets, however, NetMHCpan and NetMHC performed similarly while SMM^PMBEC^ performed worse (Additional file 1: Table S1). This is in agreement with previous performance benchmarks [9, 14, 15].

**Figure 1:**
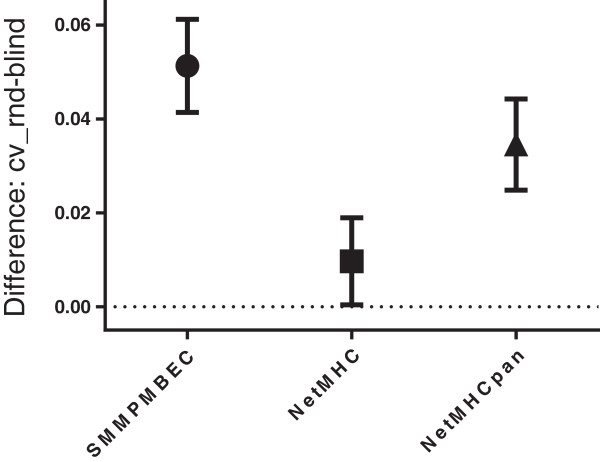
**Cross-validations tend to over-estimate predictive performances against blind data sets.** For each predictive method, a mean of its distribution of differences in performances between *cv_rnd* and *blind* (i.e. *cv_rnd - blind*) and a standard error of the mean are shown. Hence, a positive mean indicates over-estimation in performance by *cv_rnd*. Predictive performances are reported as AROC values (Area under Receiver Operating Characteristics curve). Scores of 0.5 and 1.0 indicate a random pattern and perfect prediction, respectively.

In Figure 1, standard errors of means of differences in predictive performances estimated using *cv_rnd* and performances measured on the blind data sets are shown for the three predictive methods. The means of all three distributions of prediction differences are above zero, indicating that the *cv_rnd* cross-validation strategy over-estimates blind predictive performances for all three methods. For both SMM^PMBEC^ and NetMHCpan, but not NetMHC, the over-estimation is statistically significant (t-test, two-sided), as shown in column ‘P-values: one sample’ in (see Additional file 1: Table S2) for the *cv_rnd* cross-validation strategy.

### Reducing sequence similarity in cross-validation data sets does not mitigate over-estimation of blind predictive performances

The observed over-estimations had a number of possible explanations. One previously provided explanation [13, 16] was that sequence similarity shared between training and testing data sets during cross-validation inflated predictive performances, because similar peptides are easier to predict than completely novel ones. To test this hypothesis, we utilized two cross-validation strategies that reduce sequence similarity: *cv_sr* and *cv_gs*. The *cv_sr* (cross-validation, similarity reduced) strategy reduced sequence similarity by removing peptides so that there were no similar peptides in the data sets using a sequence threshold of 80%. Once similar peptides were removed, random partitioning was done as for *cv_rnd*. In the case of the *cv_gs* strategy *(cross-validation, group similarity)*, rather than removing all similar peptides, we only required that there were no similar peptides *between* the paired training and testing sets in cross-validation partitions. Hence, in comparison to *cv_sr*, *cv_gs* kept many more peptides, but distributed them differently in the cross-validation partitions. Details of these implementations are provided in the Methods section.

**Figure 2:**
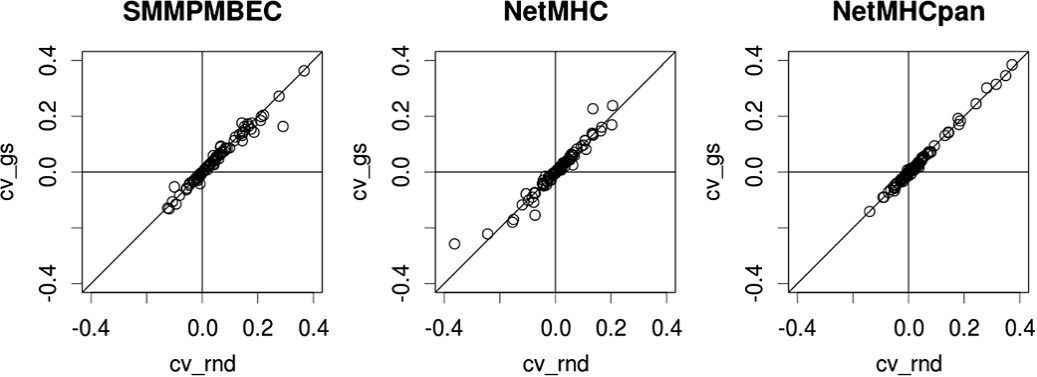
**Comparison of cross-validation strategies in terms of their differences in predictive performances with respect to those of blind.** For each strategy, its blinded performances were subtracted from those of cross-validated: *cv - blind*. Hence, positive values indicate over-estimation of blind predictive performances.

As shown in Additional file 1: Table S2, differences in AROCs between cross-validated and blind predictive performances for the cross-validation strategies *cv_sr* and *cv_gs* show means that are closer to zero than *cv_rnd* for both of these cross-validation setups. As shown in column ‘P-values: two sample’, this shift to smaller means is statistically significant for SMM^PMBEC^ and NetMHCpan, but not NetMHC. However, as shown in column ‘P-values: two-sample, absolute value’ for all three methods, using either *cv_sr* or *cv_gs* strategy did not lead to significantly more accurate estimates of blind predictive performances than *cv_rnd* (paired, one-sided, t-test). Scatter plots of deviations shown in Figure 2 confirm that *cv_rnd* and *cv_gs* perform similarly. For the remaining sections, the cross-validation strategy *cv_gs* will be used throughout.

### Data set size, evenness of peptide sequence space coverage, and range of predicted affinities can explain over-estimation of cross-validated predictive performance

**Figure 3:**
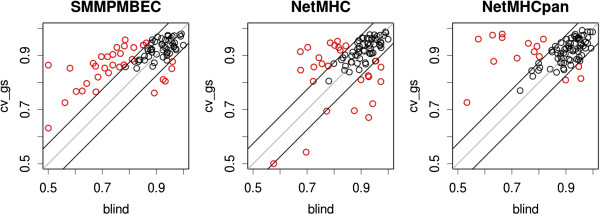
**Two classes of deviations of predictive performances estimated with cross-validation with respect to those of blind.** Predictive performances are in AROCs for the three predictive methods. The width of the band is set at values +/- mean of absolute differences between *blind* and *cv_gs* of SMM^PMBEC^: *mean(abs(cv_gs - blind)).* Data points classified as ‘large deviations’ are highlighted in red.

To better understand why predictive performances estimated using cross-validation deviated from those of blind, we defined two classes of deviations for each method, as shown in Figure 3. In the figure, a band around the diagonal differentiates cross-validated predictions with small deviations (black) from those with large deviations (red), using an arbitrary threshold defined by the mean of deviations (i.e. |cv_gs – blind|) for SMM^PMBEC^. Using the same threshold for SMM^PMBEC^, NetMHC, and NetMHCpan, 33, 26 and 15 data points were considered ‘large’ deviations, respectively.

We also looked at scatter plots of predicted versus measured binding affinities for data sets with large deviations. The scatter plots revealed that large deviations were associated with blind data sets where the ranges of predicted affinities were narrow with respect to those of cross-validated ones, or measured affinities for peptides were concentrated in the region bordering a cutoff value for binders. Additional file 1: Figure S1 is an example of the latter case for H-2 Db, where most of the peptides had measured affinities between 100 and 1000 nM, while those from the cross-validation data set were more broadly sampled (see Additional file 1).

Motivated by these observations, we characterized cross-validation and blind data sets using a number of features. Briefly, the features captured data set sizes, evenness of peptide sequence space coverage, range of predicted/measured affinities, and overlap of these ranges between cross-validation and blind data sets. The features used are listed in Table 2. Details of their calculations are provided in the Methods section.

**Table 2:** **Statistical significances of features of cross-validation and blind data sets in discriminating large deviations from small**

Features	SMM ^PMBEC^	NetMHC	NetMHCpan
log_size_cv	*7.7e-07*	*2.5e-04*	*2.5e-02*
log_size_bl	*2.9e-05*	*3.6e-03*	*1.2e-02*
entss_cv	*1.1e-04*	*1.7e-03*	*2.0e-02*
entss_bl	*3.4e-05*	*3.9e-04*	*5.1e-03*
ent_meas_cv	1.7e-01	5.5e-01	4.6e-01
ent_meas_bl	4.6e-01	5.4e-01	8.5e-01
ent_pred_cv	1.5e-01	2.1e-01	2.0e-01
ent_pred_bl	*4.8e-03*	6.4e-02	*1.1e-02*
prbol_meas	3.5e-01	9.9e-02	3.1e-01
prbol_pred	*7.8e-03*	*3.7e-02*	*2.8e-02*

Here, *deviation* = *|cv_gs - blind|,* where *blind* and *cv_gs* correspond to predictive performances in AROCs. Significant features (t-test; two-tailed at 0.05 cutoff) are italicized. See Methods for definitions of the features.

Table 2 lists features, and their statistical significances, used for discriminating cross-validated predictive performances with large deviations from those with small deviations, using the threshold introduced above. As shown, the features that showed the most significant discrimination were largely the same for all three methods. The size of the cross-validation and blind data sets (i.e. *log_size_cv* and *log_size_bl*) were among the strongest, and they inversely correlated with deviations. The next strongest features were evenness of sequence space coverage of blind and cross-validation data sets (i.e. *entss_bl* and *entss_cv*, respectively), and they also inversely correlated with deviations. The next strongest feature was the ‘spread’ of predicted affinities for the blind data sets, which also inversely correlated with deviations. For NetMHCpan, *entss_bl* was the strongest feature in discriminating the two classes of deviations, instead of data size. This difference was probably due to the fact that this pan method used data from different MHC alleles at the same time and therefore was less impacted by a low number of data points in the specific MHC allele for which predictions were made. Scatter plots of deviations versus *log_size_cv* and *entss_bl* are shown in (see Additional file 1: Figure S2).

### Logistic regression models of observed deviations for cross-validated predictive performances improve accuracy of predicting biased benchmark data sets

The results shown above suggested that certain features of the training and blind datasets could be used to identify when it is likely to observe a large difference between cross-validated and blind prediction performances. We therefore set out,to build models to quantify this likelihood using logistic regression [17], given a specific data set. Logistic regression was chosen because we wanted to model probabilities of the two classes of deviations defined earlier (i.e. large vs small) as a function of the features considered here. For each (MHC, length) combination, a logistic regression model returned a probability of large deviation based on the features for the data set, and its reference class label was based on the deviation threshold used in Figure 3. Predictive performances of the logistic regression models in AROCs were measured using leave one out cross-validations (LOOCV), where ‘testing’ set has a size of 1 while ‘training’ set the size of the remaining data.

**Figure 4:**
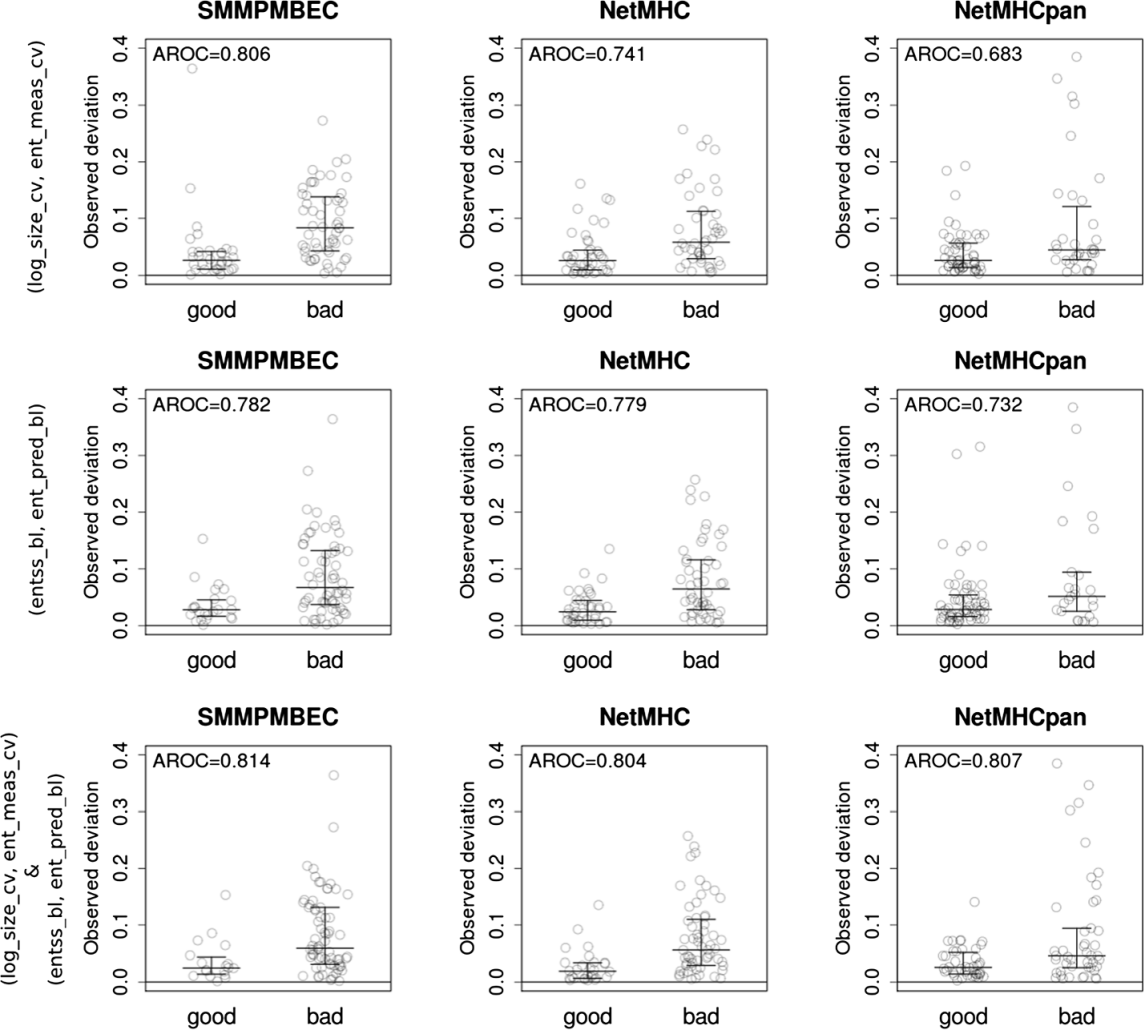
**Distributions of observed deviations for two predicted classes of deviations: small vs. large.** Leave One Out Cross-Validation (LOOCV) results were separated into two groups (i.e. ‘good’ and ‘bad’), based on a probability cutoff of 0.2. First and second rows used logistic regression models combining features indicated as row labels. Third row uses the ‘max’ approach to combine models used in the top two rows. Overlaid on top of each distribution, lower, middle, and upper line segments represent 25th, median, and 75th quartiles, respectively.

We systematically tested how well combinations of two features of the training or blind datasets could predict the likelihood of having a large deviation between cross-validated and predicted performances (see in Additional file 1: Table S3, S4 and S5). The two features of the training set with the highest predictive power were size of the dataset (*log_size_cv*) and entropy of its measured binding affinities (*ent_meas_cv*). The top row of Figure 4 shows the predictive power of a model using these features of the training set alone for SMM^PMBEC^, NetMHC and NetMHCpan methods which achieved AROC values of 0.806, 0.741 and 0.683 respectively. The predictive power of the model for NetMHCpan was likely lower as additional training data was used beyond the data available for the particular allele. After repeating the same analysis for features of the blind set, we found that a model using sequence space coverage (*entss_bl*) and entropy of the predicted binding affinities (*ent_pred_bl*) had the highest discriminatory power, achieving AROC values of 0.782, 0.779 and 0.732 for the three methods. This demonstrates that features of the blind set and features of the training set *independently* impacted the likelihood of having mismatching performance estimates between cross-validation and blind prediction performances.Finally, we built another model combining the two models described above. In this combined model, a probability of large deviation was calculated by taking a higher probability returned by the two models. The bottom row of Figure 4 shows results of LOOCV with AROC values of 0.814, 0.804 and 0.807, respectively. The combined model showed a much higher average AROC of 0.808 than the model using features of the training set (average AROC = 0.743) or blind set (average AROC = 0.764) alone, further illustrating that both training set and blind dataset need to be of adequate size and representative of the problem space in order to give consistent results in benchmarking performance.

## Discussion

To better understand how well the accuracy of peptide:MHC class I binding predictive methods can be estimated for practical applications, we utilized a large blind data set to measure the extent of deviation of cross-validated predicted performance with respect to those generated from blinded approaches. We found that cross-validations tend to over-estimate blinded prediction performance. Reducing sequence similarity between training and testing sets during cross-validation had only a marginal role in mitigating the over-estimations. Instead, a multitude of factors contributed to deviations (and over-estimations). Namely, large deviations are due to small training data sets, unevenness of sequence coverage of either training or blind data sets, and narrowly ranged predicted affinities for blind data sets.

Results from the logistic regression modeling showed that features of *both* cross-validation and blind data sets contributed to the deviations. Our results suggest that predictors trained with sufficiently large data sets that evenly cover sequence and affinity space are ‘good’, in the sense that it is less likely that they have ‘blind spots’ and will perform great on one test set but poorly on another. Based on these results, we incorporated the accuracy assessment given by the training set classifier into our benchmarking results to indicate those datasets for which the cross-validated performance measure may not be an accurate estimate (see Additional file 2).

Our results indicate that, at least for MHC-I predictors, cross-validations do give accurate estimates of ‘true’ predictive performances, if sizes of training data are sufficiently large and peptide sequence space has been sampled evenly. There are additional reasons to prefer cross-validation over blind sets: For one, it takes a long time until a sufficiently large blind dataset can be assembled to perform reliable prediction assessments. Moreover, there is a fundamental problem with blind sets being generated at least as they are submitted to the IEDB: Given the increased use of MHC binding predictions in practical applications, binding data is increasingly generated on such pre-selected peptides that will have a decreased coverage of the sequence and affinity space. As we have shown here, such blind sets are intrinsically harder to predict. Thus, the seeming drop in performance from the cross-validated predictions on the IEDB 2009 dataset compared to predictions of the data newly added into the IEDB since then is likely a reflection of the more difficult nature of these datasets.

Our study could be further improved in several ways. First, by repeating cross-validation runs with different data partitions and averaging the performance over different runs, a more solid estimate of performance could have been gathered. We did not do that in order to be directly comparable by previous benchmarks published from our group. In addition, the AROC as a performance measure is prone to generate less robust results for data sets with few numbers of binders, which is the case for several of the datasets in this study. So some of the variability between cross-validated and blind performance that we observed for datasets with low numbers of binders might be less pronounced if a different performance metric was used. However, the number of binders per dataset was one metric that was evaluated for its ability to identify datasets with highly divergent blind vs. cross-validated prediction performance, and the total number of peptides in each set in general performed better, so this issue does not seem to be a dominant concern. With these considerations, we stuck to the AROC measure to evaluate performances, also to be directly comparable to our previous benchmarks.

Others have also looked into estimating reliability of given predictions. Recently, confidence intervals of individual predictions were estimated for peptide-MHC binding based on training data [18]. We expect that such confidence interval estimations will be complementary to our findings.

## Conclusion

Using the largest ever assembled set of training and blind data sets for peptide:MHC class I binding, we determined the extent to which cross-validated predictive performances deviate from those based on blind validation. Removing sequences that shared 80% or greater sequence identity between training and testing sets during cross-validations had a marginal role in influencing the extent of deviations. Instead, we found that data set size and the evenness of coverage of the sequence space can explain most of the deviations observed for both the cross-validation and blind data set approaches. Our results identify quantitative features that will facilitate more accurate assessment of the performance of peptide-MHC binding predictors.

## Methods

### Predictive methods for binding of peptides to MHC class I molecules

Three different types of predictive methods were tested in this study. The first method was SMM^PMBEC^ [14], which is a linear regression based method and returns predictive models as position specific scoring matrices. The second method was NetMHC [19, 20], which uses neural-networks and thus may be able to capture non-linear interactions among residues. The third method was NetMHCpan [21, 22], which is also based on neural-networks. However, NetMHCpan is distinguished from NetMHC in that it is a ‘pan’ method; that is, it leverages binding data across different MHC molecules to make predictions, even for those MHCs with no previous experimental characterizations. A number of papers reporting their predictive performances have been published [9, 10, 23, 24].

### Cross-validation strategies compared

Cross-validation is a technique used to estimate accuracy of a predictive method on a single data set. This is done by first partitioning data into *N* subsets, labeling one subset as a ‘testing set’ and the remaining N-1 subsets are merged to form a ‘training set’. A prediction method is given the training set as an input and is used to make predictions against the testing set. This process is repeated as the testing set is rotated over remaining subsets. In the end, a single predictive performance of the method is calculated for the combined set of predictions made against the ‘testing’ sets.

For performance measures, Areas under Receiver Operating Characteristic curves (AROCs) [25] and Pearson’s correlation coefficients were used. In the case of AROCs, values range from 0.5 to 1.0, where 0.5 indicates random, and 1.0 perfect, predictions. An AROC value can be interpreted as the probability of distinguishing a true positive from a false positive. For the calculation of AROCs, peptides were classified into binders and non-binders at a cutoff value of 500 nM. This affinity threshold has been found to be associated with the vast majority of known T-cell epitopes [2, 26].

Different types of cross-validation strategies are possible, characterized by types of data partitioning. In this study, three different cross-validation strategies were compared. The first strategy was used in benchmarks reported in the literature, where random partitioning of data and 5-fold cross-validation were used: *cv_rnd*. The two remaining strategies involved removal of similar peptides in the data sets. One such approach, denominated ‘similarity-reduced’, has earlier been used to benchmark MHC class II predictive methods in [13]. The similarity-reduced approach deterministically removes similar peptides. This resulted in the cross-validation strategy, ‘*cv_sr’*. Another strategy is ‘*cv_gs*’, where rather than removing similar peptides entirely, similarity is removed only between testing and training partitions by grouping similar peptides in the same partition. An implementation similar to ‘*cv_gs*’ was used for benchmarking MHC-II predictive methods in [27]. Details of the two cross-validation strategies are provided in the following sections.

### Preparation of similarity reduced cross-validation data sets: *cv_sr*

To generate a similarity-reduced cross-validation data set *cv_sr* for each (MHC, length) specific data set, similar peptides were removed and data were randomly partitioned as was done for the *cv_rnd* strategy. For the removal of similar peptides, a Hobohm 1 like algorithm was used [13]. Specifically, for a given list of peptides, the peptides were sorted, from low to high, as a function of the number of ‘similar’ peptides each has. Here, two peptides were considered ‘similar’ if they shared at least 80% sequence identity and were of identical length. Starting with the peptide with the fewest neighbors (fewest number of similar peptides), the peptide was added to an initially empty set ‘*sr*’ if it was not similar to any peptide in the ‘*sr*’ set. This step was repeated until the sorted peptide set was exhausted. This approach was applied separately to binders and non-binders where binders are those with measured affinities < 500 nM and non-binders are the remaining peptides. The two sets were then combined to yield the final ‘*sr*’ set.

### Preparation of cross-validation data sets with similar peptides grouped: *cv_gs*

Rather than removing all ‘similar’ peptides, it is also possible to remove similarity only between testing and training sets, by grouping similar peptides in the same cross-validation partition. We had two additional requirements. Namely, we required that peptides were distributed across *the 5* partitions as evenly as possible. Lastly, to accommodate ‘pan’ predictive methods such as NetMHCpan that use data across alleles, we required that a peptide be assigned to the same cross-validation partition across alleles. This ensures that NetMHCpan does not gain an advantage by having similar peptides shared between testing and training sets from different alleles.

This was implemented as follows. First, peptides from the entire binding data set were clustered based on the definition of ‘similarity’ stated earlier. Specifically, peptides were represented as an undirected graph *G*, where nodes represented peptides and an edge was placed between two nodes if corresponding peptides were ‘similar’ (i.e. they shared at least 80% sequence identity and of same length). This resulted in a set of mutually exclusive subgraphs where any two nodes in each subgraph were ‘connected’ (i.e. there was a path connecting the two nodes). Each subgraph then corresponded to a cluster. Since clustering was done on all available peptides, a peptide may be associated with binding affinities measured against multiple MHC molecules.

Second, as the algorithm processed clusters from largest to smallest, it assigned one of 5 cross-validation partition indices to each cluster of peptides. For each cluster, the partition index was chosen such that peptides were distributed evenly among the 5 partitions. The selection of the partition index was achieved by identifying the MHC with the most number of measurements in the cluster and choosing the MHC’s partition index with the least number of peptides associated.

### Features of cross-validation and blind data sets

Cross-validation and blind data sets were characterized using a number of features and the following sections provide details of how the features were defined.

### Evenness of peptide sequence space coverage: *entss_cv and entss_bl*

For a set of peptide sequences, the degree of ‘evenness’ of sequence space coverage was measured using an entropy function. A discrete probability distribution function was constructed for each peptide position: *p(x*_*i*_*)*. The function describes how often a given amino acid, *x*, was found at a specific position, *i*. For each position, its entropy is defined as an expectation of the information content, *E[log(1/p(x*_*i*_*))] = -Σp(x*_*i*_*)log[p(x*_*i*_*)]* [28]. Entropies over the positions were then averaged. Higher values indicate greater degree of ‘evenness’. This measure was calculated for both cross-validation and blind data sets, resulting in features *entss_cv* and *entss_bl*, respectively. We considered using Kullback-Leibler divergence, which normalizes for an expected frequency of amino acids, but decided against it as it is not clear what background distribution should be considered here. MHC binding predictions are applied to peptides of any organism and also to artificial sequences including scans of all available amino acids; thus, we here defined entropy in such a way that all amino acids are treated equally.

### Range of binding affinities: *ent_meas_cv, ent_meas_bl, ent_pred_cv, and ent_pred_bl*

To determine the range of affinities observed for either measured or predicted data, the same entropy function used earlier was also implemented here. A probability distribution function was constructed over binned affinities, rather than 20 amino acid types as was done earlier. The binning was done for log10 transformed IC50 values, ranging from 0 to 5, with fixed bin size of 1. This measure was calculated for measured affinities of cross-validation and blind data sets (i.e. *ent_meas_cv* and *ent_meas_bl*, respectively) as well as for predicted affinities (i.e. *ent_pred_cv* and *ent_pred_bl*, respectively).

### Overlap of two ranges of affinities: *prbol_meas and prbol_pred*

To measure an overlap of ranges of two sets of affinities, the following measure was defined. Given two discrete probability distributions of log-transformed affinities defined above, *p*_*x*_*(i)* and *p*_*y*_*(i)*, a measure of their overlap is a sum of *min(p*_*x*_*(i), p*_*y*_*(i))*, where *i* indexes the bins. A higher value indicates greater overlap. The overlaps were calculated between cross-validated and blind data sets for either measured or predicted affinities, corresponding to *prbol_meas* and *prbol_pred*, respectively.

## Electronic supplementary material

Additional file 1: Figure S1: Scatter plots of measured and predicted affinities for cross-validated and blind predictions for the 9-mer data set of H-2 Db. Vertical and horizontal lines indicate cutoffs at the 500 nM threshold that distinguishes binders from non-binders. **Figure S2.** Correlations of deviation of cross-validated prediction with either data set size (i.e. log_size_cv) or entropy of sequence space (i.e. entss_bl). Here, ‘deviation’ is defined as ‘|cv_gs – blind|’. Red lines represent the class boundary used for the logistic regression modeling. **Table S1.** Average predictive performances of the three 1 methods against cv_rnd, cv_sr, cv_gs, and blind benchmark data sets as Areas under ROC curves. For each benchmark data type, highest performance is indicated with bold font. **Table S2.** Mean of differences in AROCs between predictive performances generated with cross-validations and those against blind data sets. Here, a ‘difference’ is defined as (cv – blind). Hence, positive values indicate over-estimations. In column ‘P-values: one sample’, statistical significances of over-estimations are shown (one-sided t-test). In column ‘P-values: two sample’, significances of differences in means with respect to cv_rnd for the two cross-validation strategies are shown (paired, one-sided t-tests). In column ‘P-values: two sample, absolute value’, statistical significances of improvements in estimations of blind predictive performances of either cv_sr or cv_gs with respect to cv_rnd were calculated by comparing their absolute differences (paired, one-sided t-tests). **Table S3.** Leave one out cross-validation predictive performances for each logistic regression model using a pair of features for SMMPMBEC. Performances are in AROCs. **Table S4.** Leave one out cross-validation predictive performances for each logistic regression model using a pair of features for NetMHC. Performances are in AROCs. **Table S5.** Leave one out cross-validation predictive performances for each logistic regression model using a pair of features for NetMHCpan. Performances are in AROCs. (PDF 2 MB)

Additional file 2: **A compressed file containing all predictive performances as Areas under ROC curves for the three methods.** For each method, performances were measured against the four benchmark data types: *cv_rnd*, *cv_sr*, *cv_gs*, and *blind*. Also included are probabilities of ‘large’ deviations (i.e. |*cv_gs* – *blind*|) returned by logistic regression models. (ZIP 16 KB)
