# Supplementary material for: Dataset size and composition impact the reliability of performance benchmarks for peptide-MHC binding predictions
Source: BMC Bioinformatics. 2014 Jul 14;15(1):241. doi: 10.1186/1471-2105-15-241 (PMC4111843; doi:10.1186/1471-2105-15-241)
Supplement: Supplementary file 1 — Additional file 1: Figure S1: Scatter plots of measured and predicted affinities for cross-validated and blind predictions for the 9-mer data set of H-2 Db. Vertical and horizontal lines indicate cutoffs at the 500 nM threshold that distinguishes binders from non-binders. Figure S2. Correlations of deviation of cross-validated prediction with either data set size (i.e. log_size_cv) or entropy of sequence space (i.e. entss_bl). Here, ‘deviation’ is defined as ‘|cv_gs – blind|’. Red lines represent the class boundary used for the logistic regression modeling. Table S1. Average predictive performances of the three 1 methods against cv_rnd, cv_sr, cv_gs, and blind benchmark data sets as Areas under ROC curves. For each benchmark data type, highest performance is indicated with bold font. Table S2. Mean of differences in AROCs between predictive performances generated with cross-validations and those against blind data sets. Here, a ‘difference’ is defined as (cv – blind). Hence, positive values indicate over-estimations. In column ‘P-values: one sample’, statistical significances of over-estimations are shown (one-sided t-test). In column ‘P-values: two sample’, significances of differences in means with respect to cv_rnd for the two cross-validation strategies are shown (paired, one-sided t-tests). In column ‘P-values: two sample, absolute value’, statistical significances of improvements in estimations of blind predictive performances of either cv_sr or cv_gs with respect to cv_rnd were calculated by comparing their absolute differences (paired, one-sided t-tests). Table S3. Leave one out cross-validation predictive performances for each logistic regression model using a pair of features for SMMPMBEC. Performances are in AROCs. Table S4. Leave one out cross-validation predictive performances for each logistic regression model using a pair of features for NetMHC. Performances are in AROCs. Table S5. Leave one out cross-validation predictive performances for each logist [file 12859_2014_6509_MOESM1_ESM.pdf]

Supplementary figures/tables for:

Dataset size and composition impact the reliability of performance benchmarks for peptide-MHC binding predictions

Authors

Yohan Kim[a], John Sidney[a], Soren Buus[b], Alessandro Sette[a], Morten Nielsen[c,d], Bjoern Peters[a]

[a]La Jolla Institute for Allergy & Immunology

9420 Athena Circle

La Jolla, CA 92037, USA

[b]University of Copenhagen

Department of International Health, Immunology and Microbiology

Blegdamsvej 3

2200 København N

[c]Center for Biological Sequence Analysis

Department of Systems Biology

The Technical University of Denmark

Building 208, DK-2800 Lyngby Denmark

[d]Instituto de Investigaciones Biotecnológicas,

Universidad Nacional de San Martín,

San Martín, B 1650 HMP,

Buenos Aires, Argentina

Corresponding author

Bjoern Peters

email: bpeters@liai.org

Tel: 858/752-6914

Fax: 858/752-6987

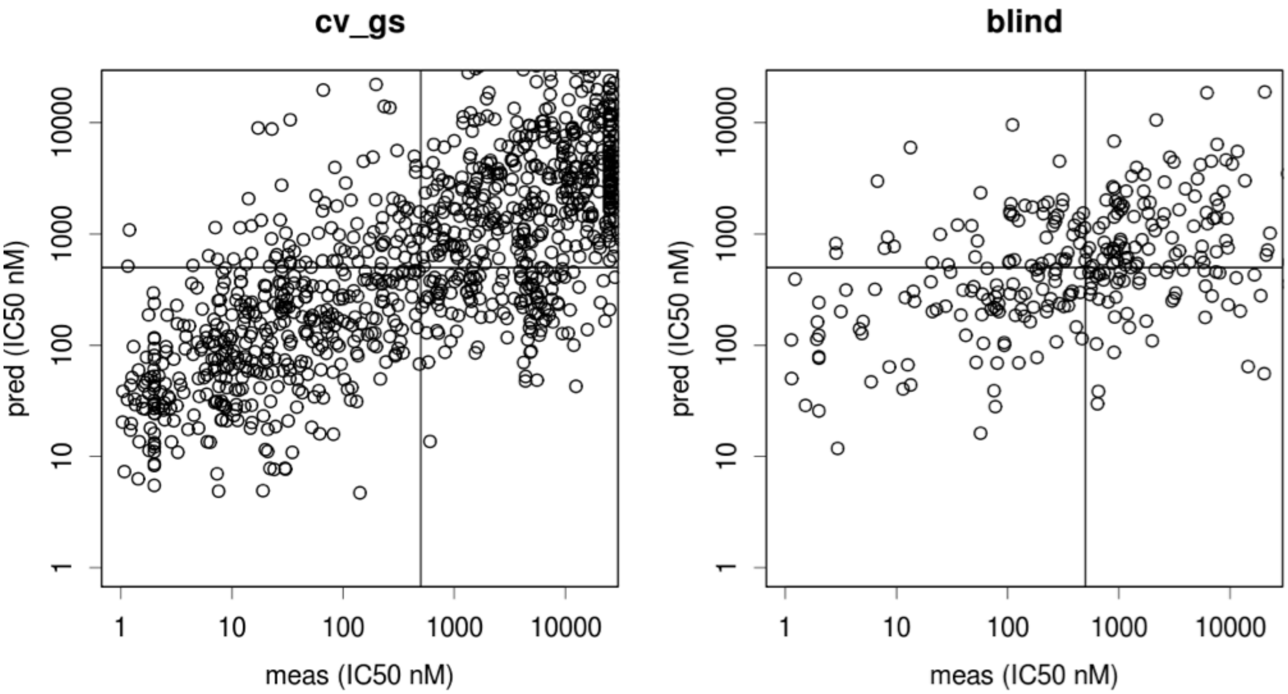

FIGURE S1. Scatter plots of measured and predicted affinities for cross-validated and blind predictions for the 9-mer data set of H-2 Db. Vertical and horizontal lines indicate cutoffs at the 500 nM threshold that distinguishes binders from non-binders.

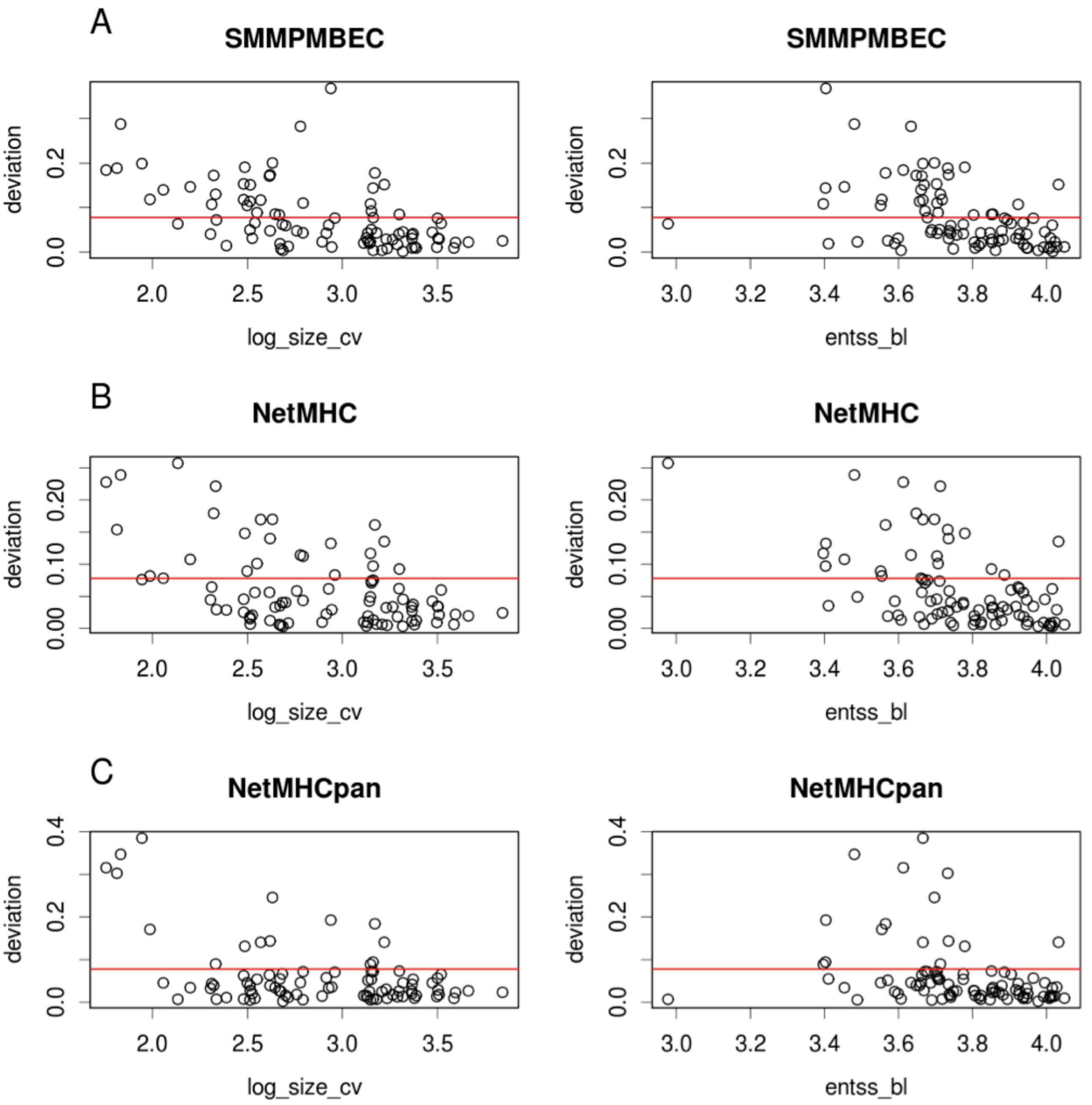

FIGURE S2. Correlations of deviation of cross-validated prediction with either data set size (i.e. log_size_cv) or entropy of sequence space (i.e. *entss_bl*). Here, 'deviation' is defined as '|*cv_gs* – *blind*|'. Red lines represent the class boundary used for the logistic regression modeling.

TABLE S1. Average predictive performances of the three methods against *cv_rnd*, *cv_sr*, *cv_gs*, and *blind* benchmark data sets as Areas under ROC curves. For each benchmark data type, highest performance is indicated with bold font.

**Method**	**cv_rnd**	**cv_sr**	**cv_gs**	**blind**
SMM^{PMBEC}	0.8989	0.8927	0.9025	0.8474
NetMHC	0.8930	0.8892	0.8919	**0.8833**
NetMHCpan	**0.9176**	**0.9149**	**0.9147**	0.8830

TABLE S2. Mean of differences in AROCs between predictive performances generated with cross-validations and those against blind data sets. Here, a 'difference' is defined as (*cv – blind*). Hence, positive values indicate over-estimations. In column 'P-values: one sample', statistical significances of over-estimations are shown (one-sided t-test). In column 'P-values: two sample', significances of differences in means with respect to *cv_rnd* for the two cross-validation strategies are shown (paired, one-sided t-tests). In column 'P-values: two sample, absolute value', statistical significances of improvements in estimations of blind predictive performances of either *cv_sr* or *cv_gs* with respect to *cv_rnd* were calculated by comparing their absolute differences (paired, one-sided t-tests).

Method	Means: (cv – blind)			P-values: one sample			P-values: two sample		P-values: two sample, absolute value	
	cv_rnd	cv_sr	cv_gs	cv_rnd	cv_sr	cv_gs	cv_rnd & cv_sr	cv_rnd & cv_gs	|cv_rnd| & |cv_sr|	|cv_rnd| & |cv_gs|
SMM^{PMBEC}	0.0513	0.0456	0.0475	1.5E-06	1.5E-05	3.0E-06	2.1E-03	2.5E-02	0.28	0.13
NetMHC	0.0097	0.0059	0.0086	3.0E-01	5.3E-01	3.5E-01	1.1E-01	3.2E-01	0.53	0.52
NetMHCpan	0.0346	0.0318	0.0317	6.0E-04	1.8E-03	2.0E-03	3.5E-03	3.8E-05	0.38	0.39

TABLE S3. Leave one out cross-validation predictive performances for each logistic regression model using a pair of features for SMM^{PMBEC}. Performances are in AROCs.

Features	log_size_cv	log_size_bl	entss_cv	entss_bl	ent_pred_cv	ent_pred_bl	ent_meas_cv	ent_meas_bl	prbol_pred	prbol_meas
**log_size_cv**	0.78	0.78	0.78	0.84	0.77	0.78	0.81	0.79	0.80	0.78
**log_size_bl**	--	0.76	0.76	0.76	0.74	0.77	0.73	0.74	0.73	0.72
**entss_cv**	--	--	0.75	0.77	0.74	0.76	0.73	0.73	0.75	0.75
**entss_bl**	--	--	--	0.78	0.78	0.78	0.76	0.78	0.77	0.77
**ent_pred_cv**	--	--	--	--	0.53	0.63	0.71	0.54	0.64	0.53
**ent_pred_bl**	--	--	--	--	--	0.66	0.73	0.68	0.69	0.64
**ent_meas_cv**	--	--	--	--	--	--	0.48	0.45	0.65	0.48
**ent_meas_bl**	--	--	--	--	--	--	--	0.31	0.62	0.43

**prbol_pred**	--	--	--	--	--	--	--	--	0.64	0.63
**prbol_meas**	--	--	--	--	--	--	--	--	--	0.47

TABLE S4. Leave one out cross-validation predictive performances for each logistic regression model using a pair of features for NetMHC. Performances are in AROCs.

**Features**	**log_size_cv**	**log_size_bl**	**entss_cv**	**entss_bl**	**ent_pred_cv**	**ent_pred_bl**	**ent_meas_cv**	**ent_meas_bl**	**prbol_pred**	**prbol_meas**
**log_size_cv**	0.73	0.75	0.76	0.83	0.71	0.73	0.74	0.73	0.73	0.74
**log_size_bl**	--	0.70	0.75	0.79	0.72	0.74	0.69	0.69	0.69	0.70
**entss_cv**	--	--	0.75	0.80	0.74	0.74	0.74	0.74	0.75	0.76
**entss_bl**	--	--	--	0.79	0.78	0.78	0.78	0.78	0.78	0.78
**ent_pred_cv**	--	--	--	--	0.49	0.57	0.67	0.54	0.59	0.59
**ent_pred_bl**	--	--	--	--	--	0.60	0.65	0.65	0.63	0.63
**ent_meas_cv**	--	--	--	--	--	--	0.39	0.29	0.58	0.54
**ent_meas_bl**	--	--	--	--	--	--	--	0.31	0.58	0.54
**prbol_pred**	--	--	--	--	--	--	--	--	0.61	0.59
**prbol_meas**	--	--	--	--	--	--	--	--	--	0.57

TABLE S5. Leave one out cross-validation predictive performances for each logistic regression model using a pair of features for NetMHCpan. Performances are in AROCs.

Features	log_size_cv	log_size_bl	entss_cv	entss_bl	ent_pred_cv	ent_pred_bl	ent_meas_cv	ent_meas_bl	prbol_pred	prbol_meas
**log_size_cv**	0.69	0.68	0.72	0.76	0.64	0.73	0.68	0.66	0.67	0.68
**log_size_bl**	--	0.62	0.72	0.72	0.64	0.71	0.60	0.59	0.59	0.59
**entss_cv**	--	--	0.73	0.76	0.72	0.75	0.72	0.72	0.72	0.75
**entss_bl**	--	--	--	0.73	0.74	0.73	0.72	0.72	0.69	0.71
**ent_pred_cv**	--	--	--	--	0.51	0.64	0.60	0.53	0.56	0.59
**ent_pred_bl**	--	--	--	--	--	0.69	0.70	0.70	0.70	0.71
**ent_meas_cv**	--	--	--	--	--	--	0.00	0.00	0.52	0.48
**ent_meas_bl**	--	--	--	--	--	--	--	0.00	0.52	0.46
**prbol_pred**	--	--	--	--	--	--	--	--	0.56	0.50
**prbol_meas**	--	--	--	--	--	--	--	--	--	0.51
